# Patterns of Transposable Element Expression and Insertion in Cancer

**DOI:** 10.3389/fmolb.2016.00076

**Published:** 2016-11-16

**Authors:** Evan A. Clayton, Lu Wang, Lavanya Rishishwar, Jianrong Wang, John F. McDonald, I. King Jordan

**Affiliations:** ^1^Integrated Cancer Research Center, School of Biology, Georgia Institute of TechnologyAtlanta, GA, USA; ^2^Ovarian Cancer InstituteAtlanta, GA, USA; ^3^School of Biology, Georgia Institute of TechnologyAtlanta, GA, USA; ^4^PanAmerican Bioinformatics InstituteCali, Colombia; ^5^Applied Bioinformatics LaboratoryAtlanta, GA, USA; ^6^Department of Computational Mathematics, Science and Engineering, Michigan State UniversityEast Lansing, MI, USA

**Keywords:** LINE-1, L1, Alu, SVA, retrotransposons, bioinformatics, mutation, tumorigenesis

## Abstract

Human transposable element (TE) activity in somatic tissues causes mutations that can contribute to tumorigenesis. Indeed, TE insertion mutations have been implicated in the etiology of a number of different cancer types. Nevertheless, the full extent of somatic TE activity, along with its relationship to tumorigenesis, have yet to be fully explored. Recent developments in bioinformatics software make it possible to analyze TE expression levels and TE insertional activity directly from transcriptome (RNA-seq) and whole genome (DNA-seq) next-generation sequence data. We applied these new sequence analysis techniques to matched normal and primary tumor patient samples from the Cancer Genome Atlas (TCGA) in order to analyze the patterns of TE expression and insertion for three cancer types: breast invasive carcinoma, head and neck squamous cell carcinoma, and lung adenocarcinoma. Our analysis focused on the three most abundant families of active human TEs: Alu, SVA, and L1. We found evidence for high levels of somatic TE activity for these three families in normal and cancer samples across diverse tissue types. Abundant transcripts for all three TE families were detected in both normal and cancer tissues along with an average of ~80 unique TE insertions per individual patient/tissue. We observed an increase in L1 transcript expression and L1 insertional activity in primary tumor samples for all three cancer types. Tumor-specific TE insertions are enriched for private mutations, consistent with a potentially causal role in tumorigenesis. We used genome feature analysis to investigate two specific cases of putative cancer-causing TE mutations in further detail. An Alu insertion in an upstream enhancer of the *CBL* tumor suppressor gene is associated with down-regulation of the gene in a single breast cancer patient, and an L1 insertion in the first exon of the *BAALC* gene also disrupts its expression in head and neck squamous cell carcinoma. Our results are consistent with widespread somatic activity of human TEs leading to numerous insertion mutations that can contribute to tumorigenesis in a variety of tissues.

## Introduction

More than 50% of the human genome sequence is derived from transposable element (TE) insertions (Lander et al., 2001; de Koning et al., 2011). The vast majority of TE-derived sequences in the human genome correspond to relatively ancient insertions that are no longer capable of transposition (Mills et al., 2007). However, there are several families of human TEs that remain active to this day. The most abundant families of active TEs in the human genome are the Alu and SVA short interspersed nuclear elements (SINEs) along with the L1 Long Interspersed Nuclear Element (LINE) family (Kazazian et al., 1988; Batzer and Deininger, 1991; Batzer et al., 1991; Brouha et al., 2003; Ostertag et al., 2003; Wang et al., 2005). Alu and SVA SINEs are non-autonomous TEs that are mobilized via the transpositional machinery encoded by the autonomous L1 family of LINEs. Recent evidence indicates that a handful of HERV-K endogenous retroviral elements also remain active in the human genome (Wildschutte et al., 2016).

Active TE families are of great interest since they have the ability to generate *de novo* mutations, many of which have been linked to human disease (Hancks and Kazazian, 2012; Solyom and and Kazazian, 2012). For instance, TE insertions have been shown to contribute to the etiology of a variety of different cancer types (Belancio et al., 2010a; Carreira et al., 2014). Numerous recent studies have used a combination of next-generation sequence analysis, followed by validation with PCR and/or Sanger sequencing, to elucidate connections between TE activity and cancer (Solyom et al., 2012; Shukla et al., 2013; Tubio et al., 2014; Doucet-O'Hare et al., 2015; Ewing et al., 2015). L1 insertions in particular have been implicated as potential cancer causing mutations in those and other studies (Morse et al., 1988; Miki et al., 1992; Iskow et al., 2010; Lee et al., 2012; Scott et al., 2016). L1 activity is thought to promote tumor development by causing genomic instability, via impaired chromosomal pairing during mitosis, and/or by disrupting coding or regulatory sequences (Kemp and Longworth, 2015).

Many of the studies that have related TEs to cancer have considered TE expression, at the transcript or protein level, and TE insertional activity separately. A number of different cancer types are positive for L1 transcript expression (Belancio et al., 2010b), and L1 proteins have been shown to be ubiquitously expressed in both normal and tumor samples from the same individuals (Bratthauer and Fanning, 1992, 1993; Bratthauer et al., 1994; Asch et al., 1996; Doucet-O'Hare et al., 2015, 2016). There is also evidence suggesting that L1 protein expression can be limited to tumor tissues and thereby serve as a useful cancer biomarker; nearly half of all human cancers are exclusively immunoreactive for L1-ORF1 encoded proteins (Rodic et al., 2014). The expression of L1 proteins in tumors has been shown to affect the expression of a number of cancer-related genes, including the down-regulation of tumor suppressors (Rangasamy et al., 2015). With respect to TE insertional activity, studies on matched normal and tumor tissues have found that novel L1 insertions occur at high frequencies in lung cancer genomes (Iskow et al., 2010). Such insertions frequently occur in oncogenes and tumor suppressors, underscoring their putative role in tumorigenesis (Lee et al., 2012).

A principal challenge when interpreting cancer genomes is distinguishing between so-called passenger and driver mutations. While passenger mutations are present in cancer genomes, they are not considered to contribute to cancer progression; instead, they are simply somatic mutations that arise during carcinogenesis and are carried along during clonal expansion. Driver mutations, on the other hand, are causal mutations that are directly implicated in carcinogenesis and the promotion of cancer growth (Stratton et al., 2009; Marx, 2014; Pon and Marra, 2015). To date, only a few studies have directly implicated TE insertions as cancer driver mutations. One such study analyzed 19 hepatocellular carcinoma genomes utilizing the RC-Seq methodology (Baillie et al., 2011) and discovered two separate L1 insertions that initiate tumorigenesis via distinct oncogenic pathways (Shukla et al., 2013). This study found L1 insertions in two different tumor suppressor genes: Mutated in Colorectal Cancers (*MCC*) and Suppression of Tumorigenicity (*ST18*). Most recently, a role for L1 insertional activity was conclusively demonstrated for colorectal cancer caused by an insertion in the *APC* tumor suppressor gene (Scott et al., 2016). This paper describes a somatic L1 insertion into one copy of the *APC* gene that, when coupled with a point mutation in the other copy of the gene, initiates tumorigenesis through the two hit colorectal cancer pathway.

**Figure 1:**
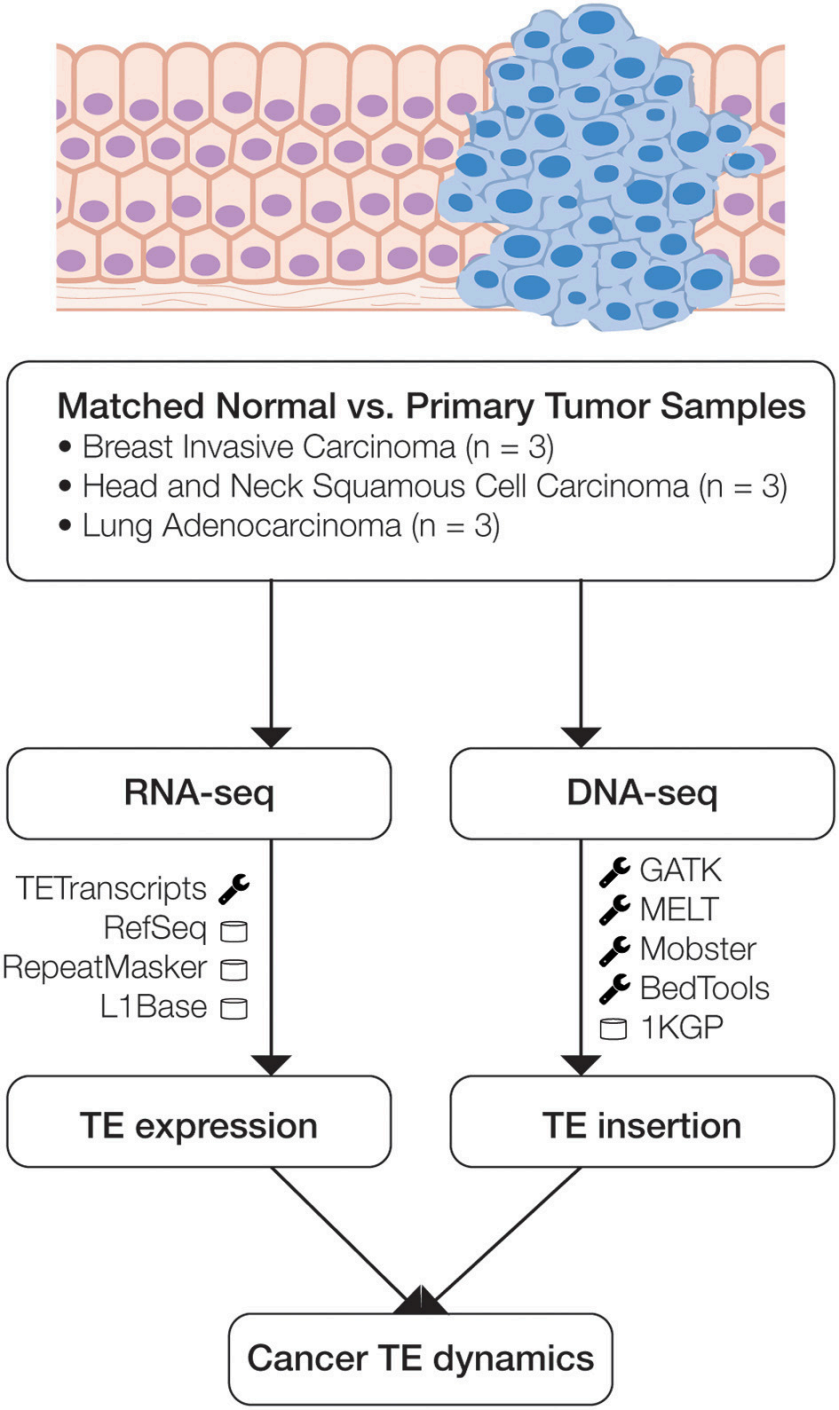
**Scheme of the analytical design used in this study**. Matched normal and primary tumor samples for three cancer types were analyzed using transcriptome (RNA-seq) and whole genome (DNA-seq) data. RNA-seq data was analyzed to compare normal versus cancer expression levels, and DNA-seq data was analyzed to identify somatic TE insertion events. The main bioinformatics programs (wrench) and databases (cylinder) used for each phase of the analysis are indicated.

Owing to parallel developments in genomics and bioinformatics, it is now possible to jointly analyze the patterns of TE transcript expression and TE insertional activity in human cancers. The Cancer Genome Atlas (TCGA) provides access to both transcriptome sequence data (RNA-seq) and whole genome sequence data (DNA-seq) for a number of matched normal and primary tumor sample pairs from individual patients (Weinstein et al., 2013). In addition, recently developed bioinformatics algorithms allow for the detection of TE transcripts directly from RNA-seq data (Jin et al., 2015) as well as for the characterization of novel TE insertions from DNA-seq data (Thung et al., 2014; Sudmant et al., 2015). We took advantage of these developments in order to evaluate the patterns of both TE expression and insertional activity in three cancer types: breast invasive carcinoma, head, and neck squamous cell carcinoma, and lung adenocarcinoma (Figure 1 and Supplementary Figure 1). We observed a simultaneous increase of L1 transcript expression and L1 insertional activity for primary tumor samples for all three cancers, and we evaluate individual cases of TE insertions that are implicated as potential cancer causing mutations.

## Materials and methods

### Genome and transcriptome sequence data

Whole genome sequence data (DNA-seq), transcriptome sequence data (RNA-seq) and patient metadata for matched normal and primary tumor tissue samples from nine cancer patients were acquired from The TCGA (Weinstein et al., 2013) via the Cancer Genomics Hub (CGHub) using the download client GeneTorrent (Maltbie et al., 2013). The nine participants included three breast invasive carcinoma patients, three head and neck squamous cell carcinoma patients and three lung adenocarcinoma patients (Table 1). DNA-seq and RNA-seq data were accessed as BAM files of paired-end Illumina sequence data aligned against the human genome reference sequence (build hg19). BAM files containing sequence alignments were validated for quality using FASTQC (Andrews, 2011), and autosomes were extracted from the BAM files for downstream analysis using SAMtools (Li et al., 2009).

**Table 1:** **TCGA whole genome (DNA-seq) and transcriptome (RNA-seq) data sources for the patients analyzed in this study**.

**ID**	**TCGA barcode**	**Cancer type**	**Sex**	**Age**	**Sample type^a^**	**Seq depth**	**Read len**.
Breast 1	TCGA-BH-A0B3-11B-21D-A128-09	Breast invasive carcinoma	F	53	NT-W	42.4	100
	TCGA-BH-A0B3-11B-21R-A089-07				NT-R	5.5	50
	TCGA-BH-A0B3-01A-11D-A128-09				TP-W	40.2	100
	TCGA-BH-A0B3-01B-21R-A089-07				TP-R	5.4	50
Breast 2	TCGA-BH-A0BW-11A-12D-A314-09		F	71	NT-W	54.1	100
	TCGA-BH-A0BW-11A-12R-A115-07				NT-R	7	50
	TCGA-BH-A0BW-01A-11D-A10Y-09				TP-W	46.1	100
	TCGA-BH-A0BW-01A-12R-A115-07				TP-R	7.3	50
Breast 3	TCGA-BH-A0DT-11A-12D-A12B-09		F	41	NT-W	63.3	100
	TCGA-BH-A0DT-11A-12R-A12D-07				NT-R	7.7	50
	TCGA-BH-A0DT-01A-21D-A12B-09				TP-W	79.9	100
	TCGA-BH-A0DT-01A-21R-A12D-07				TP-R	6.6	50
Head 1	TCGA-CV-7255-11A-01D-2276-10	Head and neck squamous cell carcinoma	F	32	NT-W	6.9	101
	TCGA-CV-7255-11A-01R-2016-07				NT-R	7.5	48
	TCGA-CV-7255-01A-11D-2276-10				TP-W	5.8	101
	TCGA-CV-7255-01A-11R-2016-07				TP-R	7.1	48
Head 2	TCGA-CV-7416-11A-01D-2334-08		F	29	NT-W	7.7	101
	TCGA-CV-7416-11A-01R-2081-07				NT-R	5.9	48
	TCGA-CV-7416-01A-11D-2334-08				TP-W	28.6	101
	TCGA-CV-7416-01A-11R-2081-07				TP-R	6	48
Head 3	TCGA-CV-6959-11A-01D-1911-02		M	48	NT-W	38.3	51
	TCGA-CV-6959-11A-01R-1915-07				NT-R	8.5	48
	TCGA-CV-6959-01A-11D-1911-02				TP-W	31.4	51
	TCGA-CV-6959-01A-11R-1915-07				TP-R	6.6	48
Lung 1	TCGA-44-6776-11A-01D-1853-02	Lung adenocarcinoma	F	60	NT-W	38.9	51
	TCGA-44-6776-11A-01R-1858-07				NT-R	5.4	48
	TCGA-44-6776-01A-11D-1853-02				TP-W	6.9	51
	TCGA-44-6776-01A-11R-1858-07				TP-R	7.4	48
Lung 2	TCGA-50-5932-11A-01D-1753-08		M	75	NT-W	34.6	101
	TCGA-50-5932-11A-01R-1755-07				NT-R	4.2	48
	TCGA-50-5932-01A-11D-1753-08				TP-W	44.5	101
	TCGA-50-5932-01A-11R-1755-07				TP-R	7.4	48
Lung 3	TCGA-55-6984-11A-01D-1945-08		F	NA	NT-W	36.2	101
	TCGA-55-6984-11A-01R-1949-07				NT-R	4.9	48
	TCGA-55-6984-01A-11D-1945-08				TP-W	41	101
	TCGA-55-6984-01A-11R-1949-07				TP-R	5.2	48

a *NT-D, Normal tissue DNA-seq; NT-R, Normal tissue RNA-seq; TP-D, Tumor primary DNA-seq; TP-R, Tumor primary RNA-seq*.

### Gene and transposable element (TE) expression levels

Gene and TE expression levels were measured using RNA-seq data for the nine matched normal and primary tumor tissue samples. Gene expression levels were quantified as read counts mapped to NCBI RefSeq gene annotations (Pruitt et al., 2012). TE expression levels—for Alu, L1 and SVA elements—were quantified using reads mapped to RepeatMasker annotations, which were subsequently analyzed with the TEtranscripts package (Jin et al., 2015). The TEtranscripts program uses an expectation maximization (EM) algorithm to choose optimal unique TE locations for multi-mapped reads, thereby allowing for accurate expression level measurements for active TE families. The TEtranscripts method was recently shown to yield more reliable measures of TE transcription levels compared to previously published methods, such as HTSeq-count, Cufflinks, and RepEnrich (Trapnell et al., 2010; Criscione et al., 2014; Anders et al., 2015). The L1Base database was used to identify the genomic locations of 145 full length, intact elements from the most recently active L1 subfamily (Penzkofer et al., 2005). The set of full-length intact L1 sequences from the L1Base was generated by performing a BLAST search using the human genomic DNA sequences against the L1 template sequence (Penzkofer et al., 2005). L1Base was used to facilitate measures of active L1 element expression by limiting our analysis to RNA-seq reads that map to full-length, intact L1 sequences which retain the potential to be transpositionally active. This was done in an effort to ensure that the reads we analyzed were taken from potentially active L1 elements as opposed to older fixed elements, which could represent read-through transcripts initiated from nearby genomic promoters. The expression levels of these potentially active L1 elements were analyzed separately using the TEtranscripts method.

Differential expression levels between normal and cancer tissue pairs, for genes and TEs, were evaluated by comparing distributions of log_10_ transformed RNA-seq expression levels characterized as described above. The statistical significance levels of the observed differential expression between normal and cancer pairs were evaluated by comparing these distributions using the non-parametric Kolmogorov-Smirnov test. Statistical comparisons were done separately for each tissue (cancer) type: breast invasive carcinoma, head and neck squamous cell carcinoma and lung adenocarcinoma.

### Transposable element insertion detection

The genomic locations of novel TE insertions from matched normal and primary tumor tissue samples were predicted based on discordant read-pair mapping of DNA-seq data (Ewing, 2015) (Table 2). A scheme of our TE insertion detection analysis pipeline is shown in Supplementary Figure 2. DNA-seq BAM files were realigned according to GATK's standard indel realignment method (Van der Auwera et al., 2013) to facilitate TE insertion detection. The programs MELT (Sudmant et al., 2015) and Mobster (Thung et al., 2014) were used together for TE insertion detection. These two programs were selected owing to their previously demonstrated superior performance for human TE insertion detection (Rishishwar et al., 2016). Only TE insertion sites that were found by both methods (i.e., the intersection of the predictions) were used for subsequent analysis. TE insertion predictions made by the individual programs were considered to represent the same insertion if they were found within ±100 bp of each other. An additional filtering step was applied based on the number of mapped sequence reads (coverage) that support each TE insertion prediction. Only predictions with a minimum coverage of 5 reads and a maximum coverage of 4X the average sequencing depth of the sample were used for subsequent analysis. These upper and lower cut-off thresholds were empirically chosen based on the observed distributions of the numbers of discordant mapped read pairs used to call individual TE insertions. Read count distributions were computed individually for each program (MELT, Mobster) used and for each sample (Supplementary Figure 3). The resulting distributions were typically bimodal with a lower peak (i.e., with lower read count support) that we considered to be enriched for potential false positive TE insertion calls. The lower cut-off threshold of 5 reads was chosen to minimize such false positives, and the upper cut-off threshold was chosen to remove calls made in genomic regions that show anomalously high numbers of mapped reads, which tend to be enriched for ambiguously mapped reads.

**Table 2:** **Numbers of MELT and Mobster predicted TE insertions in matched normal (N) and primary tumor (T) samples across 9 individuals**.

**Participant ID**	**TE insertions in matched normal tissue**				**TE insertions in tumor primary tissue**			
	**Alu**	**SVA**	**L1**	**Total**	**Alu**	**SVA**	**L1**	**Total**
Breast 1	913	28	127	1069	853	33	110	997
Breast 2	1004	21	121	1147	1160	54	143	1358
Breast 3	1012	63	139	1215	952	60	136	149
Head 1	984	72	140	1197	741	66	107	915
Head 2	945	25	131	1102	832	26	138	997
Head 3	860	36	108	1005	819	41	112	973
Lung 1	716	29	92	838	780	36	113	930
Lung 2	806	25	103	935	701	20	94	816
Lung 3	856	21	110	988	746	14	100	861

The number of observed versus expected counts of unique L1 insertions were compared for matched normal and primary tumor tissue samples. The observed counts were taken from the TE detection pipeline, and the expected counts were computed as the ratio of unique insertions seen in matched normal vs. primary tissue for all TEs multiplied by the total number of observed L1 insertions. The significance of the difference between the observed versus expected counts of unique L1 insertions was evaluated using the Fisher's exact test. Counts of TE insertions for matched normal and primary tumor tissue samples were characterized based on their frequencies from the 1000 Genomes Project (1KGP) (Sudmant et al., 2015) and grouped into three distinct frequency bins. The distributions of TE insertion counts across the three frequency bins were compared for matched normal and cancer samples for the different tissue types analyzed here, and the significance of the differences between these distributions were evaluated using the Kolmogorov-Smirnov test.

### TE insertion genome feature analysis

The genomic locations of novel TE insertions were considered with respect to several genomic features using the BEDTools program (Quinlan, 2014): RefSeq genes (Pruitt et al., 2012), COSMIC tumor suppressor genes (Forbes et al., 2015), and enhancer elements defined by chromatin states (Roadmap Epigenomics et al., 2015). The population allele frequencies of the predicted TE insertions were computed from the Phase 3 release of the 1KGP (Sudmant et al., 2015) as previously described (Rishishwar et al., 2015).

## Results and discussion

### TE expression levels in matched normal vs. primary tumor tissue samples

**Figure 2:**
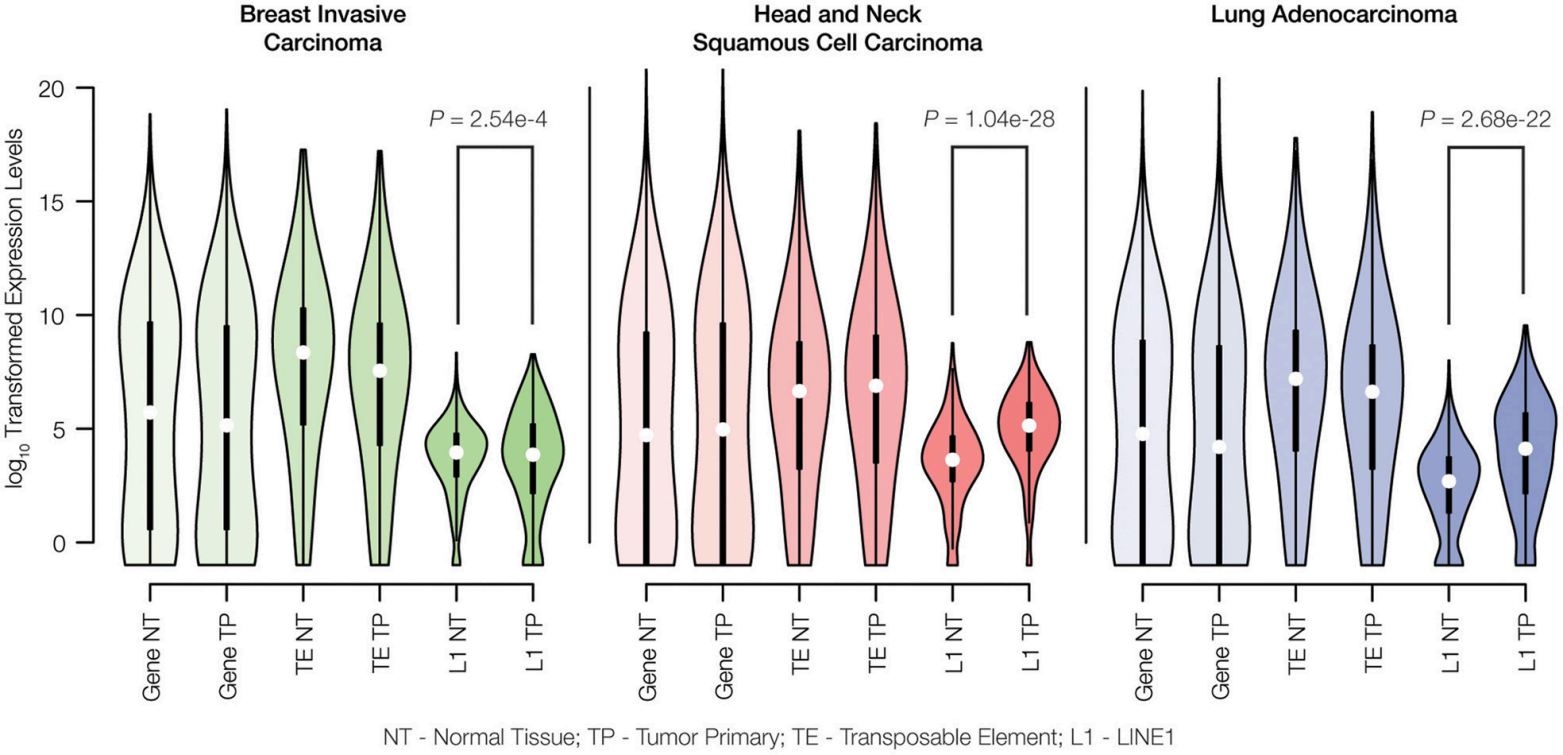
**Gene expression levels for matched normal vs. primary tumor tissue pairs**. Normal tissue (NT) and tumor primary (TP) expression levels were measured for genes, transposable elements (TEs) and LINE1 elements (L1s) via analysis of RNA-seq data as described in the Materials and Methods. Expression levels are shown as distributions of log_10_ transformed read counts, and normal versus tumor comparisons are shown for breast invasive carcinoma (green), head and neck squamous cell carcinoma (red), and lung adenocarcinoma (blue). For each tissue type, the significance levels of the differences in L1 expression between normal and cancer pairs are indicated with *P*-values from the Kolmogorov-Smirnov test.

RNA-seq data were used to evaluate the differences in TE expression levels between matched normal and primary tumor tissue samples as described in the Materials and Methods. The observed differences in gene expression levels between normal and tumor tissue were compared to differences in TE expression levels for breast invasive carcinoma, head, and neck squamous cell carcinoma and lung adenocarcinoma. There are no significant differences observed for the distributions of gene expression levels between matched normal and primary tumor tissue pairs for any of the three cancer types analyzed here (Figure 2). Similarly, when all three families of potentially active TEs (Alu, L1, and SVA) are considered together, there is no significant difference seen for the overall levels of expression between matched normal and tumor tissue. However, when full-length, potentially active L1 sequences are considered alone, we observe statistically significant increases in L1 expression levels for all three cancer types.

The methods that we used to characterize TE expression levels include several analytical controls aimed to ensure that only genuine TE-initiated transcripts, from members of potentially active families, are measured. Nevertheless, the lack of a difference between normal and tumor expression levels observed when all three active TE families were considered together could reflect technical difficulties with identifying *bona fide* TE transcripts that are initiated from element promoters as opposed to TE sequences that are passively expressed as part of longer genic transcripts. This is particularly true for Alu elements, many of which are found in the introns of human genes and transcribed as read-through transcripts initiated from RNA Pol II gene promoters (Deininger, 2011). Our confidence in the ability to measure L1-initiated transcripts is higher owing to the focus on previously identified full-length, intact elements that are located in intergenic regions. In any case, the up-regulation of L1s in cancer that we observed has potential implications for increased TE insertional activity for all three families, since L1 encoded proteins are responsible for the *cis* retrotransposition of L1s as well as the *trans* activation of Alu and SVA elements (Batzer and Deininger, 2002; Hancks and Kazazian, 2010). We analyzed the same pairs of matched normal and primary tumor tissues to evaluate whether the observed increase in L1 expression corresponds to increased transpositional activity of human TEs.

### Novel TE insertions in matched normal and primary tumor tissue samples

It is now possible to characterize the genomic locations and copy numbers of individual TE insertions from whole genome DNA-seq data owing to recent developments in computational genomics software (Ewing, 2015; Rishishwar et al., 2016). This technological advance is exemplified by the recent Phase 3 release of the 1KGP, which includes a complete genome-wide census of polymorphic TE insertion sites for 2504 individuals across 26 human populations (Sudmant et al., 2015). We analyzed whole genome DNA-seq data using computational methods for TE insertion detection (see Materials and Methods) in order to compare TE insertional activity between matched normal versus primary tumor tissue samples.

**Figure 3:**
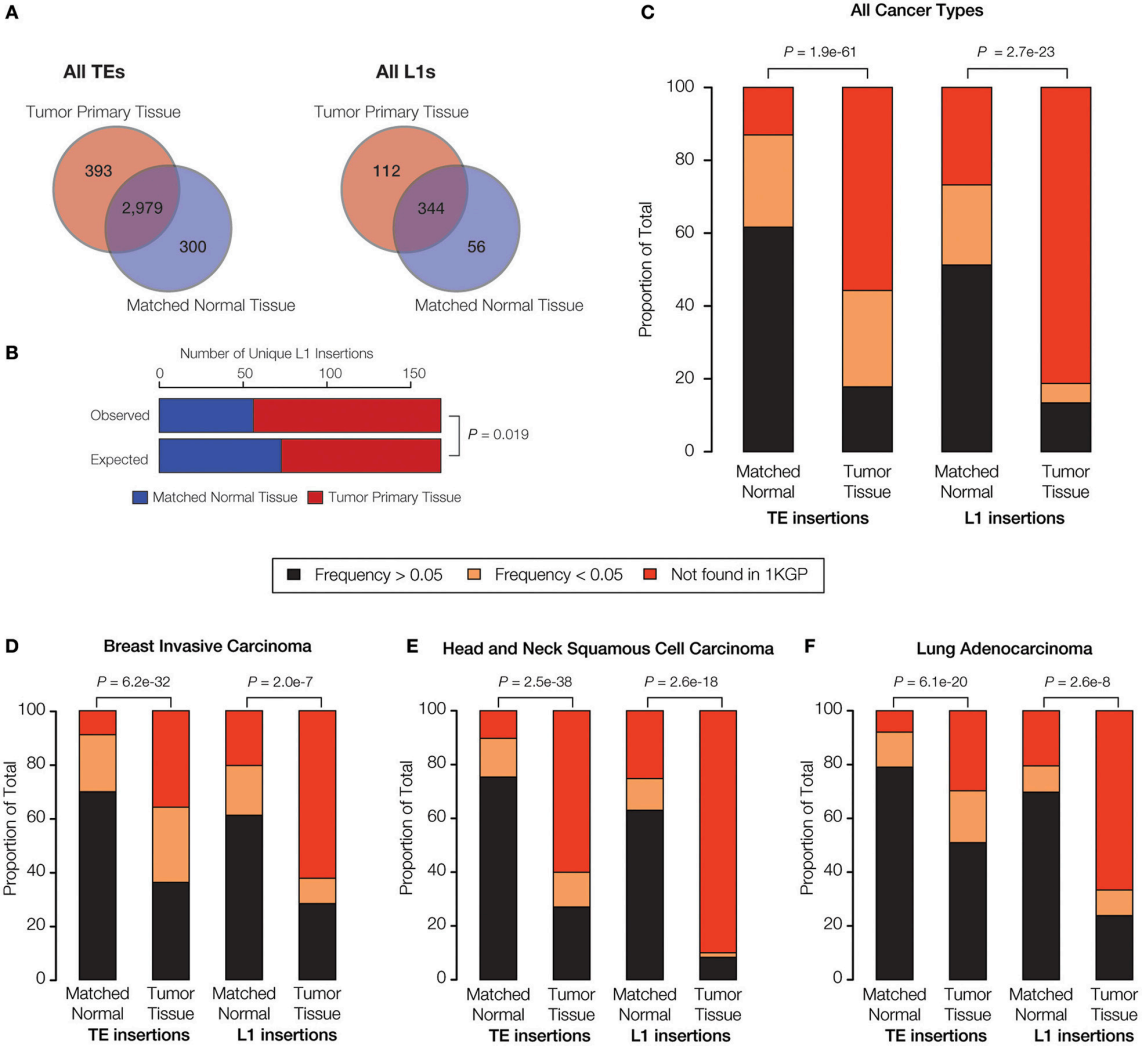
**TE insertional activity in matched normal vs. primary tumor tissue pairs**. The number of TE insertions were measured for normal and primary tumor tissue pairs for breast invasive carcinoma, head, and neck squamous cell carcinoma and lung adenocarcinoma via analysis of whole genome DNA-seq data as described in the Materials and Methods. **(A)** The total number of predicted TE insertions, pooled for all nine individuals over the three cancer types analyzed here, are shown for normal vs. tumor tissue. Venn diagrams show the numbers of unique versus shared TE insertions for the two tissue types. **(B)** Comparison of the observed versus expected numbers of unique L1 insertions for normal vs. tumor tissue. **(C)** Comparison of the population frequencies of observed TE insertions in matched normal vs. tumor tissue pairs are shown for all of the TEs analyzed here and for L1s alone. **(D–F)** The same comparisons of TE insertion population frequencies are shown individually for each cancer type analyzed here. TE insertion population frequencies are color coded as shown in the key. *P*-values show the significance of the differences for observed distributions based on the Fisher's exact test **(B)** and the Kolmogorov-Smirnov test **(C–F)**.

When all three families of active human TEs are considered together, we observed a total of 3672 TE insertions across the nine individuals analyzed for normal and cancer tissue pairs, 693 of which are unique insertions found in only one individual and one tissue type. In other words, we observe an average of ~77 unique somatic TE insertions per person, i.e., “private” TE insertions. This estimate is similar to the value of ~90 unique (presumably germline) TE insertions that we previously observed for individuals from the 1KGP (Rishishwar et al., 2015). A large majority of the observed TE insertions—81% for all TEs and 62% for L1s alone—are shared between the normal and tumor tissue types of an individual, suggesting that they represent germline insertions (Figure 3A). There are 1.3x more unique TE insertions seen for tumor compared to normal tissue, and this effect is more pronounced for L1s alone, which are 2x more abundant in tumor tissue samples. Accordingly, there is a statistically significant excess of observed versus expected L1 insertions in tumor versus normal tissue (*P* = 0.019) (Figure 3B). These results are consistent with a potential role for L1 transpositional activity in tumorigenesis for the cancer types analyzed here, as has been previously suggested for several different cancers (Morse et al., 1988; Iskow et al., 2010; Lee et al., 2012; Scott et al., 2016).

Given the relatively high level of L1 insertional activity in the tumor tissue samples analyzed here, we tested whether tumor-specific L1 insertions are found at lower frequencies among the (presumably) healthy donors from the 1KGP compared to L1 insertions found in matched normal tissue. The idea was to evaluate whether the tumor-specific L1 insertions represent mutations that are private, and thereby more likely to be deleterious or disease-causing. To do this, individual TE insertions were classified as high frequency (>0.05), low frequency (<0.05) or private (absent) according to their previously characterized population (allele) frequencies from the 1KGP (Rishishwar et al., 2015; Sudmant et al., 2015).

When all three cancer types are considered together, there is a statistically significant excess of private and low frequency TE insertions observed for tumor compared to normal tissue (*P* = 1.9e-61) (Figure 3C). This effect is even more pronounced when L1 insertions are considered alone (*P* = 2.7e-23). The same pattern of an increased frequency of private L1 insertions in tumor tissue is observed (*P* < 2.0e-7) when all three cancer types are analyzed for sets of patients (Figures 3D–F) and when samples for individual patients are analyzed separately (Supplementary Figure 4). The strongest effect is seen for head and neck squamous cell carcinoma. The pattern of a significant excess of private L1 insertions in tumor compared to normal tissue, observed for all three cancer types studied here, provides further evidence in support of a possible role for L1 activity in tumorigenesis.

It should be noted TE insertions found in low copy numbers may not be detectable using next-generation sequence analysis, whereas such insertions may be uncovered using more sensitive PCR-based approaches. False negatives of this kind will be more prevalent at low levels of sequence coverage. We have tried to control for this by using relatively high sequence coverage (~35X) studies here, but the conservative lower read count cut-off of 5 reads per TE insertion call that we used may still lead to missing TE insertion calls. Sequence based predictions can also yield false-positive TE insertion calls. In an effort to deal with this issue, we have only used high-confidence calls produced by two independent programs—MELT and Mobster—that we have recently shown to be most reliable for the detection of human TE insertions (Rishishwar et al., 2016).

One other potential problem with the sequence based analysis relates to the base pair resolution with which TE insertions can be called via computational analysis of next-generation sequence data. Currently, the most accurate programs for calling TE insertions from next-generation sequence data do not yet allow for the insertions to be precisely located to genomic regions at single base pair resolution. To account for this fact, TE insertions called within a window of ±100 bp are considered to be co-located (Supplementary Figure 2). It is possible that this approximation can lead to multiple TE insertion events being collapsed into a single event. Subsequent experimental confirmation of individual TE insertion calls of interest (e.g., potentially tumorigenic TE insertions) should help to provide certainty with respect to both their validity and their precise genomic locations.

### Potentially tumorigenic TE insertions

Having established a potential role for transpositional activity in tumorigenesis using the genome-wide approaches described above, we wanted to search for specific examples where individual TE insertions could be implicated as possible cancer driver mutations. To do so, we performed an integrated analysis of TE insertion, gene expression and chromatin data (see Materials and Methods) in an effort to identify the cancer-specific TE insertions that are most likely to play a causal role in tumorigenesis. We considered TE insertions that are co-located with either exons or regulatory elements of previously characterized tumor suppressor genes to have the highest likelihood of being functionally relevant. We observed a total of 141 intragenic (35.9%) insertions and 246 intronic insertions (62.6%) out of the 393 total cancer-specific insertions in our dataset. None of these intergenic or intronic cancer-specific TE insertions were found to disrupt any known functional (regulatory) sequence element. Thus, consistent with previous studies, the vast majority of TE insertions that we observed are not likely to affect gene function or expression in cancer. We did find 4 exonic TE insertions, along with 2 insertions located in regulatory elements, for known tumor suppressor genes (1.5% of the total). Here, we focus on two of these potential cases of cancer driver TE insertions, which could prove to be of interest to the TE and/or cancer research communities.

**Figure 4:**
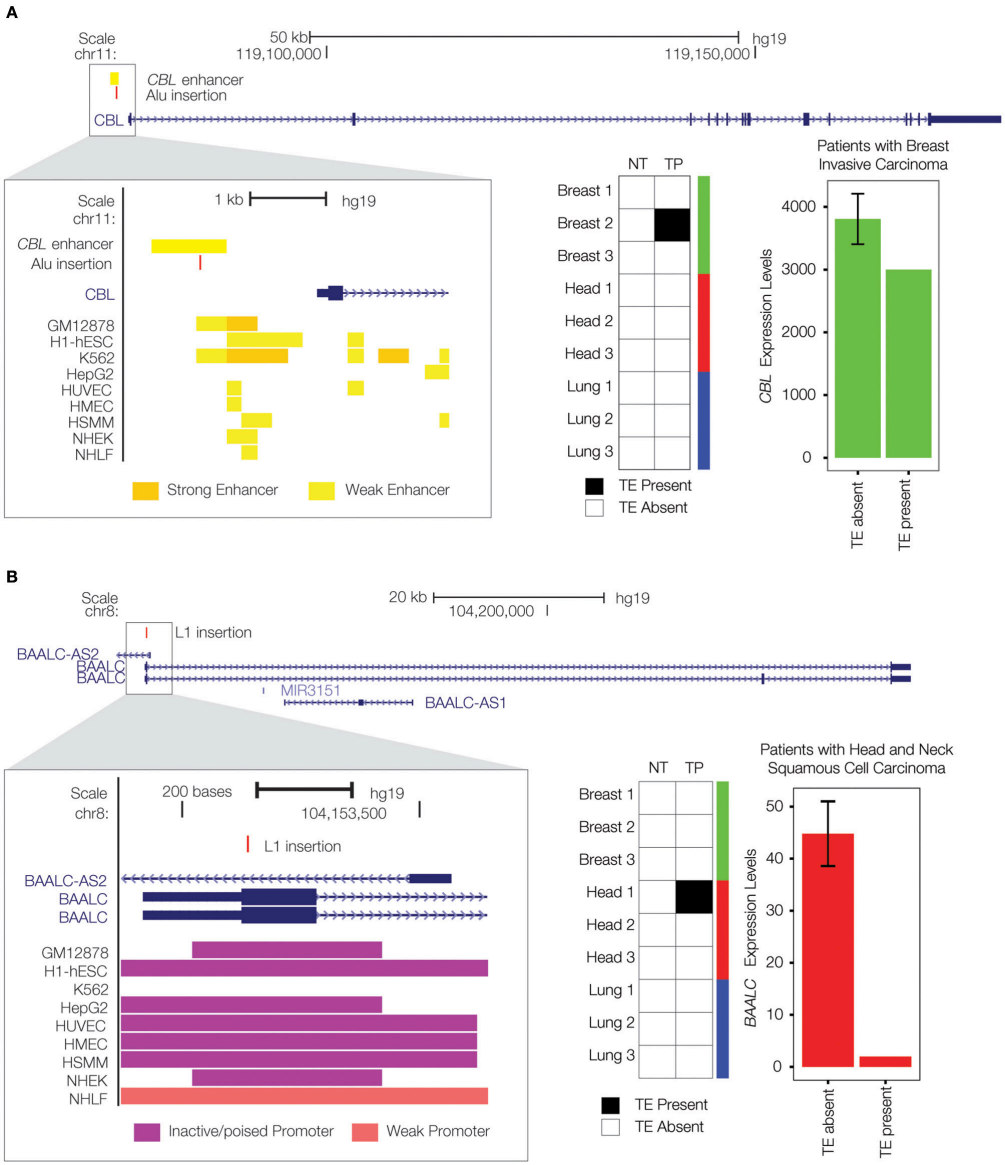
**Private TE insertions implicated as potential cancer driver mutations. (A)** A tumor-specific Alu insertion (red) is found in a single breast cancer patient. The insertion is located within an upstream enhancer for the *CBL* gene on chromosome 11 (gene model shown in blue), as indicated by enhancer-associated chromatin marks (inset yellow bars). Presence of the Alu insertion is associated with down-regulation of *CBL* (expression levels in green). **(B)** A tumor-specific L1 insertion (red) is located within the first exon of the *BAALC* gene on chromosome 8 (gene model shown in blue). Co-location of the L1 insertion with promoter-associated chromatin marks (purple bars) is shown in the inset. Presence of the L1 insertion is associated with down-regulation of *BAALC* (expression levels in red).

There is a private, breast cancer tumor-specific Alu insertion that is located within an upstream enhancer element that helps to regulate the expression of the Cbl Proto-Oncogene (*CBL*) gene (Figure 4A). *CBL* is classified as a tumor suppressor gene by the COSMIC database (Forbes et al., 2015). It has been found to be mutated or translocated in a number of cancers including acute myeloid leukemia (Abbas et al., 2008; Naramura et al., 2011; Aranaz et al., 2013); mutations in *CBL* are also the cause of Noonan syndrome-like disorder (Martinelli et al., 2010). The *CBL* encoded protein functions as a negative regulator of signal transduction pathways (Schmidt and Dikic, 2005), activation of which have been associated with cancer (Sever and Brugge, 2015). The tumor-specific Alu enhancer insertion that we characterized is associated with down-regulation of *CBL* expression, consistent with a potential role in tumorigenesis via the activation of signal transduction pathways associated with cell proliferation (Sever and Brugge, 2015).

We also found a private L1 insertion that was unique to a head and neck squamous cell carcinoma tissue sample, located within the first exon of the Brain and Acute Leukemia, Cytoplasmic (*BAALC*) gene (Figure 4B). As its name implies, the *BAALC* gene is expressed in the brain and related neural tissues, and it was first identified by association with acute myeloid leukemia where it was shown to be overexpressed (Damiani et al., 2013; Zhou et al., 2015). TE insertions within exons are extremely rare and would presumably have a dramatic effect on gene function. Indeed, this particular insertion is associated with nearly complete inactivation of the *BAALC* gene. This is consistent with previous results showing that the presence of fixed L1 insertions genome-wide is strongly associated with the down-regulation of human gene expression (Han et al., 2004). A recent study has demonstrated that *BAALC* can inhibit extracellular signal-regulated kinase (ERK) mediated monocytic differentiation of AML cells (Morita et al., 2015). Thus, down-regulation of *BAALC* would presumably result in a loss of control over cellular differentiation, consistent with a possible role in tumorigenesis. A recent study discovered a role for the change in methylation status of a cancer-specific L1 insertion in tumorigenesis (Scott et al., 2016); this could be an additional mechanism by which the *BAALC* L1 insertion observed here exerts a regulatory effect.

## Conclusion

The results of our analysis show a surprisingly high level of somatic TE activity in the human genome. Abundant transcripts from members of all three active human TE families analyzed here—Alu, SVA and L1—can be identified for both normal and cancer tissue samples. In addition, after filtering for high confidence TE insertion calls, we identified an average of close to 80 unique insertions for each tissue among the individual patients in our study. Thus, active human TE families retain the ability to transpose in somatic tissue thereby generating substantial levels of cellular heterogeneity among diverse tissues.

We also observe a correlated increase in both transcript expression levels and transpositional activity for L1 elements in cancer tissue samples when compared to matched normal tissue. Increased cancer expression of L1 elements is particularly relevant for TE insertional activity, since the L1 transpositional machinery is responsible for transposing non-autonomous Alu and SVA elements in *trans* along with L1 elements in *cis*. Our results are consistent with previous studies showing expression of L1 transcripts in lung cancer (Belancio et al., 2010b) and expression of L1 ORF1p in breast cancer (Harris et al., 2010), and tumor-specific L1 insertions have also previously been found in breast (Morse et al., 1988), head and neck (Helman et al., 2014), and lung tumors (Helman et al., 2014). We confirmed the presence of numerous tumor-specific L1 insertions in these three cancer types and identify two potentially tumorigenic TE insertions, an Alu insertion in the enhancer region of the tumor suppressor gene *CBL* and an L1 insertion in the first exon of the *BAALC* gene. These results underscore the potential for somatic TE activity to generate cellular heterogeneity and to contribute to the etiology of cancer across a wide range of human tissues.

## Ethics statement

Ethical approval was not required for this study on restricted access, de-identified data in accordance with the guidelines of the Cancer Genome Atlas (TCGA). Access to the data was approved by the data access committee of the TCGA.

## Author contributions

EC, LW, and LR performed all of the analyses described in the study. JW contributed to the genome feature analysis. IJ and JM conceived of designed and supervised the study. All authors contributed to the drafting and revision of the manuscript.

## Funding

EC and LW were supported by the Georgia Tech Bioinformatics Graduate Program. LR and IJ were supported by the IHRC-Georgia Tech Applied Bioinformatics Laboratory (ABiL).

### Conflict of interest statement

The authors declare that the research was conducted in the absence of any commercial or financial relationships that could be construed as a potential conflict of interest.
