# Supplementary material for: Patterns of Transposable Element Expression and Insertion in Cancer
Source: Front Mol Biosci. 2016 Nov 16;3:76. doi: 10.3389/fmolb.2016.00076 (PMC5110550; doi:10.3389/fmolb.2016.00076)
Supplement: Supplementary file 1 [file Image1.pdf]

Supplementary Material

Dynamics of transposable element expression and insertion in cancer

Evan A. Clayton, Lu Wang, Lavanya Rishishwar, Jianrong Wang, John F. McDonald and I. King Jordan*

*** Correspondence:** Corresponding Author: king.jordan@biology.gatech.edu

1 Supplementary Figures

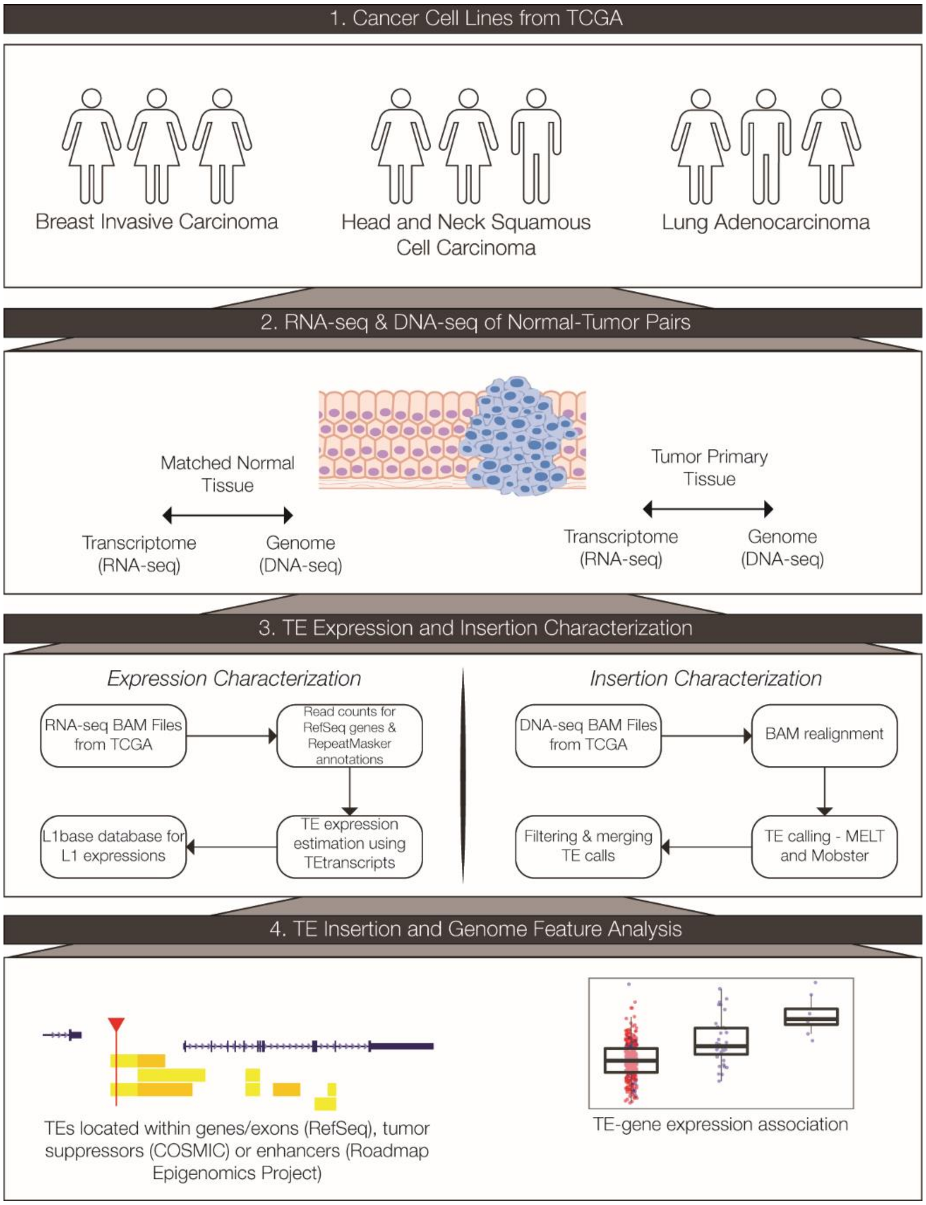

Supplementary Figure 1. **Scheme of the analysis pipeline used for this study.** 1) Matched normal and primary tumor sample data for three patients each from three cancer types were obtained from TCGA. 2) Transcriptome (RNA-seq) and whole genome sequence (DNA-seq) data were compared for normal versus tumor tissue samples. 3) RNA-seq and DNA-seq data were analyzed to characterize TE expression levels and TE insertional activity for normal versus tumor tissue samples as shown. 4) Genomic features associated with tumor-specific TE insertions were evaluated to look for putative TE cancer causing mutations.

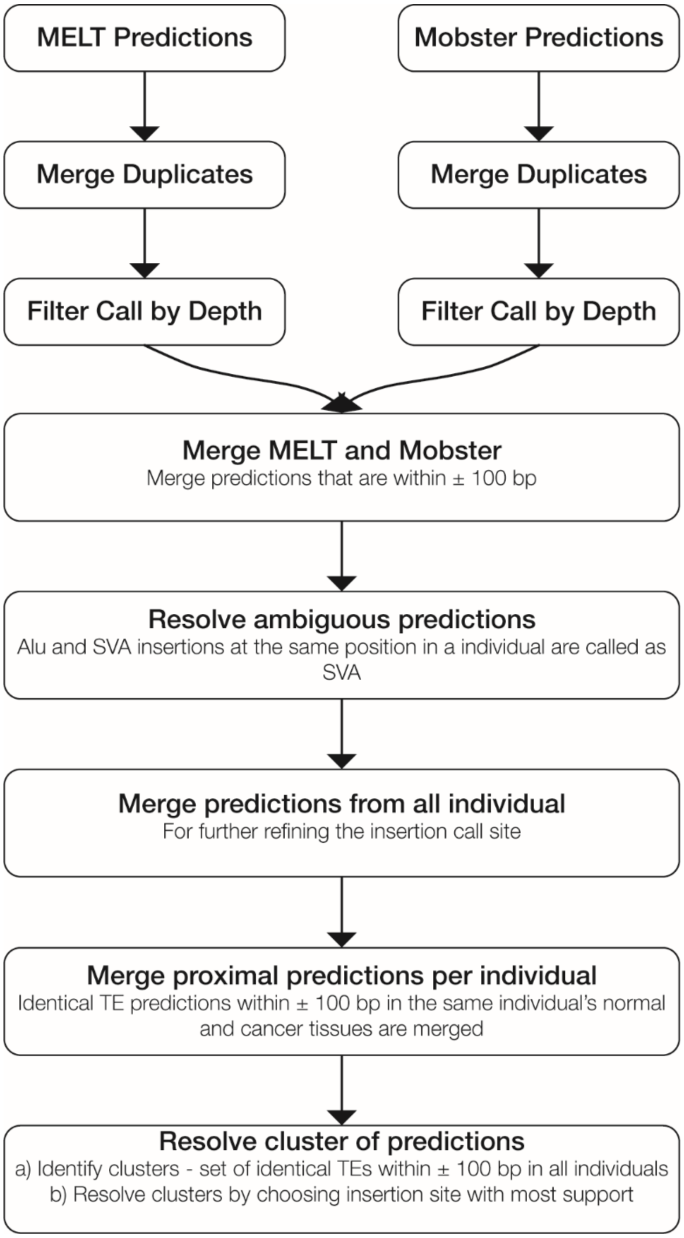

Supplementary Figure 2. **Scheme of the TE insertion detection analysis pipeline used for this study**. Steps used to merge predictions from the MELT and Mobster programs are shown along with the post-processing steps used to ensure that accurate TE insertion predictions were chosen for subsequent analysis.

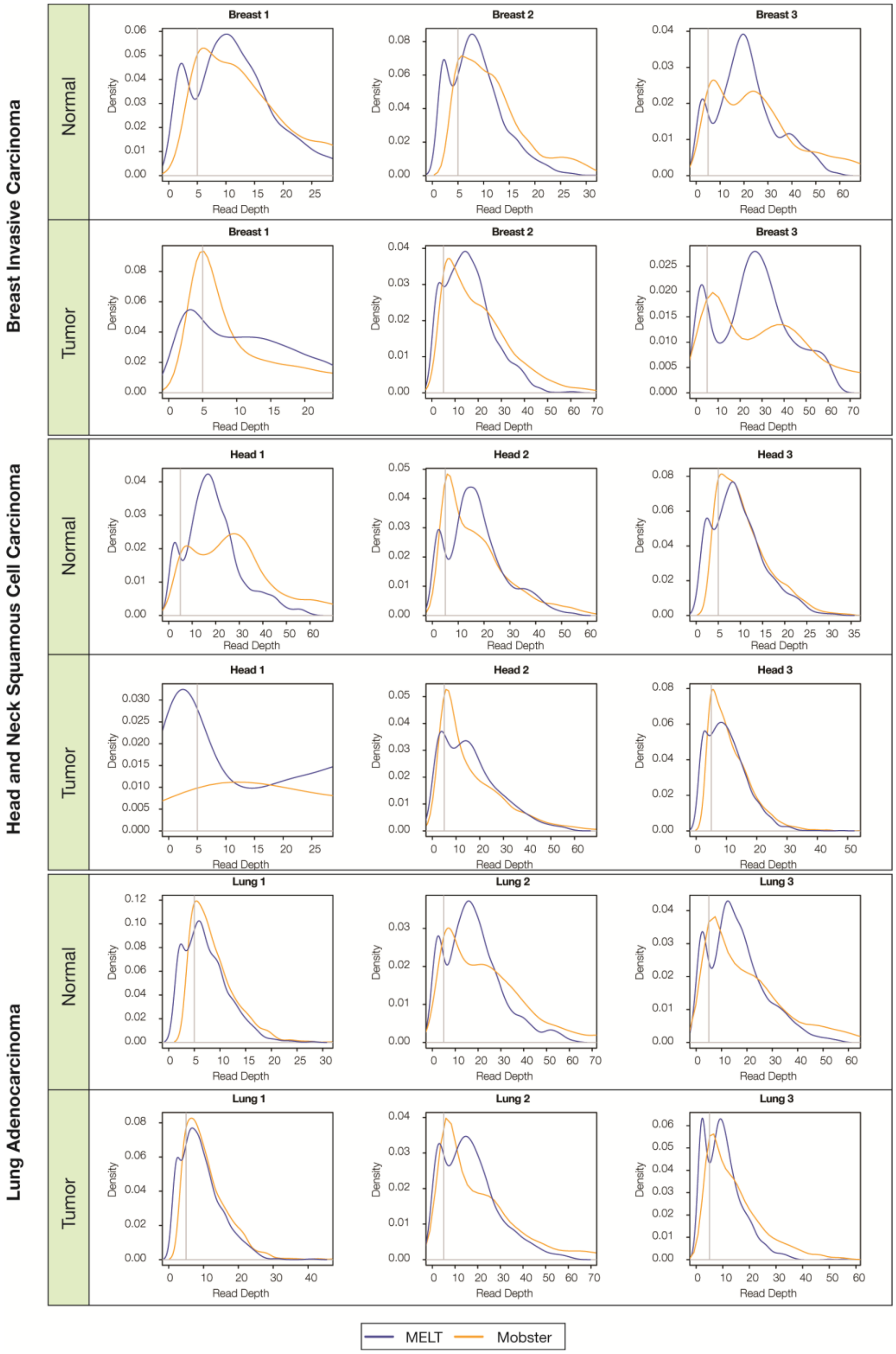

Supplementary Figure 3. **Density distributions for the numbers of mapped reads supporting TE insertion calls.** Read depth distributions are shown for TE insertion calls made with the MELT (blue) and Mobster (orange) programs for all 18 of the matched normal and primary tumor tissue samples analyzed here. The locations of the lower read depth threshold of 5 reads are indicated for each distribution with a gray line, and the distributions are all bounded by the upper read depth threshold corresponding to 4X the average sequencing depth of the sample.

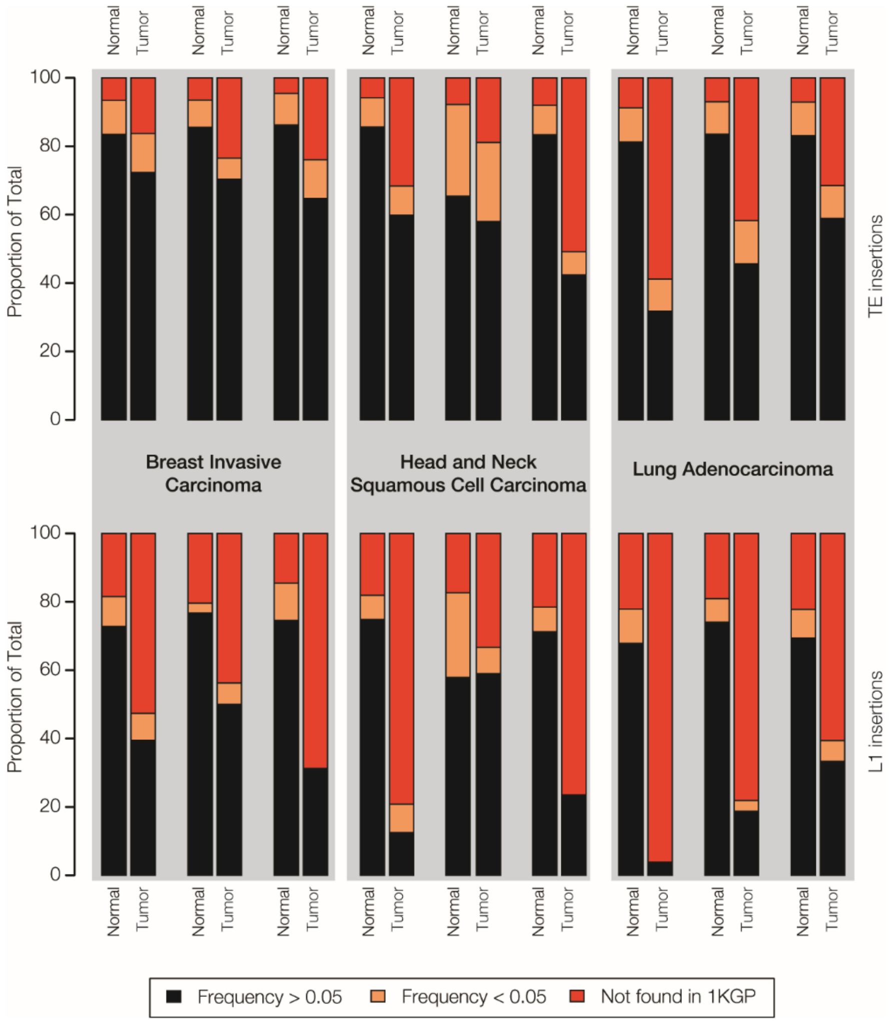

Supplementary Figure 4. **Population frequencies of observed TE insertions in matched normal versus tumor tissue pairs are shown for all of the TEs analyzed here and for L1s alone.** Frequencies are presented for the same three cancer data sets analyzed here, as shown in Figure 3, but the data here are shown for each individual sample across the three cancer types.
